# Personalized swallowing rehabilitation program for post-surgical dysphagia in oral cancer patients: a randomized controlled trial

**DOI:** 10.3389/fonc.2026.1809479

**Published:** 2026-04-22

**Authors:** Yanhong Zhang, Hua Chen, Jie Zhou, Nannan Li

**Affiliations:** Department of Oral and Maxillofacial Head and Neck Oncology, Shanghai Fengcheng Hospital, Shanghai, China

**Keywords:** dysphagia, free flap reconstruction, oral cancer, quality of life, randomized controlled trial, swallowing rehabilitation

## Abstract

**Background:**

Dysphagia following oral cancer ablative surgery with free flap reconstruction significantly impairs quality of life, yet personalized rehabilitation approaches remain understudied. This randomized controlled trial compared a personalized swallowing rehabilitation program with standard care in these patients.

**Methods:**

This single-center randomized controlled trial enrolled 300 patients with oral squamous cell carcinoma who underwent tumor resection with free flap reconstruction between January 2022 and December 2025 at Shanghai Fengcheng Hospital. Patients were randomly assigned (1:1) to receive either a personalized swallowing rehabilitation program (n=150) incorporating neuromuscular electrical stimulation, surface electromyography biofeedback, tongue pressure resistance training, and individualized exercise protocols, or standard care (n=150) consisting of conventional swallowing exercises. The primary outcome was the Functional Oral Intake Scale (FOIS) score at 6 months. Secondary outcomes included MD Anderson Dysphagia Inventory (MDADI) scores, aspiration rates, time to oral feeding recovery, feeding tube dependency, and aspiration pneumonia incidence, assessed at 1, 3, and 6 months postoperatively.

**Results:**

At 6 months, the personalized rehabilitation group demonstrated significantly higher FOIS scores compared with standard care (median, 7 [interquartile range (IQR), 6–7] vs 6 [IQR, 5–6]; Hodges-Lehmann median difference, 1.00; 95% CI, 1.00–1.00; P < 0.001; effect size r = 0.366). The intervention group showed superior MDADI composite scores (median, 75.57 [IQR, 65.99–86.06] vs 65.80 [IQR, 56.30–74.40]; P < 0.001), lower aspiration rates (12.0% vs 21.3%; relative risk, 0.56; P = 0.044), shorter time to oral feeding recovery (median, 15.84 vs 19.80 days; P < 0.001), and reduced feeding tube dependency at 6 months (8.7% vs 28.7%; P < 0.001). Subgroup analyses demonstrated consistent benefits across tumor sites, clinical stages, and reconstruction types, though the effect was attenuated in patients receiving adjuvant chemoradiotherapy (P = 0.201). The mean adherence rate in the intervention group was 78.96% ± 13.34%.

**Conclusions:**

A personalized swallowing rehabilitation program significantly improves functional swallowing outcomes, reduces aspiration risk, and enhances swallowing-related quality of life compared with standard care in oral cancer patients following surgical resection with free flap reconstruction. These findings support the integration of individualized, multimodal rehabilitation strategies into routine postoperative management.

## 1 Introduction

Oral squamous cell carcinoma represents a significant global health burden, with approximately 377,000 new cases diagnosed annually worldwide (1). Surgical resection remains the cornerstone of treatment for resectable disease, frequently necessitating ablative procedures followed by microvascular free flap reconstruction to restore anatomical integrity and function (2). Despite advances in reconstructive techniques, post-surgical dysphagia persists as a prevalent and debilitating complication, affecting 50% to 80% of patients undergoing extensive oral cavity resection (3–5).

The pathophysiology of post-surgical dysphagia in oral cancer patients is multifactorial, encompassing structural alterations from tissue resection, sensory deficits from nerve sacrifice, and motor impairment from muscular disruption (6). Free flap reconstruction, while essential for wound closure and functional rehabilitation, introduces additional bulk and altered tissue compliance that may further compromise swallowing biomechanics (7). These anatomical and physiological changes manifest clinically as impaired bolus formation, reduced tongue propulsion, delayed pharyngeal swallow initiation, and inadequate airway protection, collectively increasing aspiration risk and compromising nutritional intake (8).

The consequences of untreated or inadequately managed post-surgical dysphagia extend beyond nutritional compromise. Aspiration pneumonia, a leading cause of morbidity and mortality in this population, occurs in 15% to 25% of patients within the first year following treatment (9). Furthermore, swallowing dysfunction profoundly impacts psychological well-being and social functioning, with patients reporting diminished quality of life related to eating difficulties, social isolation during meals, and anxiety regarding aspiration events (10–12).

Current evidence supporting swallowing rehabilitation in head and neck cancer populations has primarily focused on patients receiving radiotherapy or chemoradiotherapy, with the landmark PRESTO trial and subsequent studies demonstrating the benefits of prophylactic swallowing exercises (13–15). However, surgical patients present distinct rehabilitation challenges, as their dysphagia stems from immediate anatomical disruption rather than progressive radiation-induced fibrosis (16). The optimal rehabilitation approach for this population remains inadequately defined, with existing protocols often employing standardized exercise regimens that fail to account for individual variations in resection extent, reconstruction type, and residual functional capacity (17).

Emerging rehabilitation modalities offer promising adjuncts to conventional swallowing exercises. Neuromuscular electrical stimulation (NMES) has demonstrated efficacy in enhancing swallowing muscle strength and coordination in various dysphagia populations, though evidence in post-surgical oral cancer patients remains limited (18–20). Surface electromyography (sEMG) biofeedback facilitates motor learning by providing real-time visual feedback of muscle activation patterns, enabling patients to optimize their swallowing effort (21). Tongue pressure resistance training addresses the specific deficit of reduced lingual propulsive force commonly observed following glossectomy procedures (22). Integration of these modalities within a personalized framework that tailors intervention intensity and focus to individual patient characteristics represents a potentially superior approach to standardized protocols.

This randomized controlled trial was designed to evaluate the efficacy of a personalized swallowing rehabilitation program compared with standard care in oral cancer patients following surgical resection with free flap reconstruction. We hypothesized that the personalized intervention, incorporating multimodal rehabilitation techniques tailored to individual patient deficits, would result in superior functional swallowing outcomes, reduced aspiration rates, and improved swallowing-related quality of life compared with conventional rehabilitation approaches.

## 2 Methods

### 2.1 Study design and oversight

This single-center, parallel-group, randomized controlled trial was conducted at Shanghai Fengcheng Hospital between January 2022 and December 2025. The study protocol was approved by the Ethics Committee of Shanghai Fengcheng Hospital (Approval No.: FC2022159). All participants provided written informed consent prior to enrollment. The trial was conducted in accordance with the Declaration of Helsinki and reported following the Consolidated Standards of Reporting Trials (CONSORT) guidelines.

### 2.2 Participants

**Figure 1:**
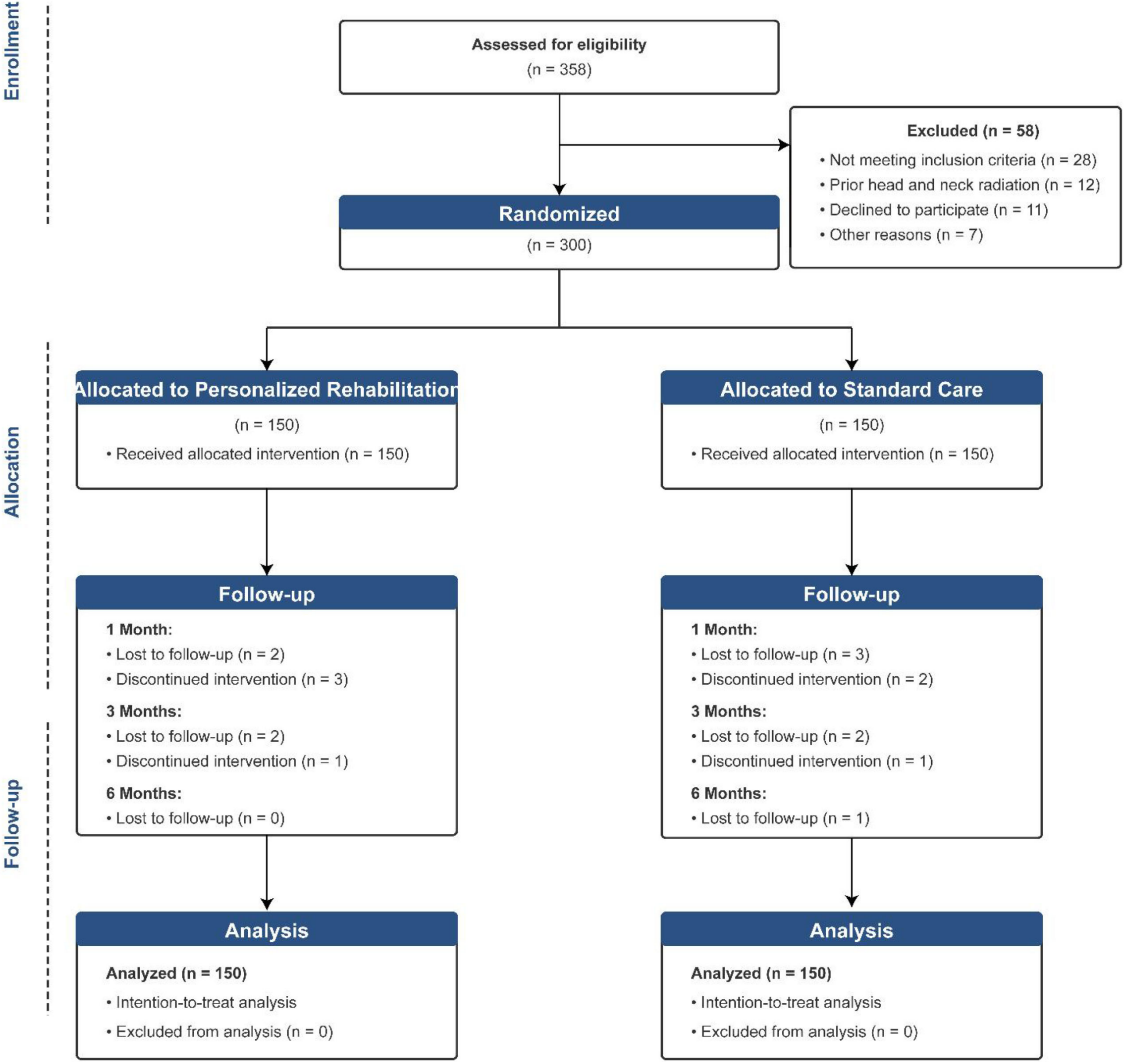
CONSORT flow diagram showing patient enrollment, randomization, and follow-up.

Eligible patients were adults (age ≥18 years) with histologically confirmed oral squamous cell carcinoma scheduled to undergo primary tumor resection with microvascular free flap reconstruction. Inclusion criteria comprised clinical stages I-IV disease amenable to surgical resection, Eastern Cooperative Oncology Group performance status 0-2, and ability to comprehend and comply with rehabilitation protocols. Exclusion criteria included prior head and neck radiation therapy, pre-existing neurological conditions affecting swallowing, uncontrolled systemic disease precluding participation in rehabilitation, and cognitive impairment limiting protocol adherence (**Figure 1**).

### 2.3 Randomization and blinding

Following baseline assessment on postoperative day 7, eligible patients were randomly assigned in a 1:1 ratio to receive either personalized swallowing rehabilitation or standard care. Randomization was performed using a computer-generated sequence with permuted blocks of variable sizes (4, 6, and 8), stratified by tumor site (tongue vs other) and clinical stage (I-II vs III-IV). Treatment allocation was concealed using sequentially numbered, opaque, sealed envelopes. Due to the nature of the intervention, blinding of patients and treating therapists was not feasible; however, outcome assessors were blinded to group allocation.

### 2.4 Interventions

#### 2.4.1 Personalized swallowing rehabilitation program

The intervention comprised a multimodal, individualized rehabilitation protocol initiated on postoperative day 7 and continued for 6 months. The program consisted of four core components tailored to each patient’s specific deficits as identified through clinical swallowing evaluation and videofluoroscopic swallowing study performed at baseline. The total daily training time was approximately 60–90 minutes, depending on the specific combination of exercises prescribed for each patient.

First, neuromuscular electrical stimulation was applied using a portable stimulation device (VitalStim Plus, Chattanooga Group) with electrodes positioned on the submental and laryngeal regions. Stimulation parameters (frequency 30–80 Hz, pulse width 300 μs, intensity at sensory threshold to maximum tolerated level) were individualized based on patient tolerance and therapeutic response. Sessions were conducted for 30 minutes daily during the inpatient period, transitioning to home-based therapy 5 days weekly following discharge.

Second, surface electromyography biofeedback training utilized a portable sEMG device with electrodes placed on the submental muscle group. Patients performed effortful swallows while observing real-time visual feedback of muscle activation amplitude, with targets set at 50% to 75% above baseline resting levels. Biofeedback sessions comprised 30 repetitions performed three times daily.

Third, tongue pressure resistance training employed the Iowa Oral Performance Instrument (IOPI) to strengthen lingual musculature, consistent with established strength-training principles for dysphagia rehabilitation (22, 23). Patients performed anterior and posterior tongue elevation exercises against resistance, with intensity set at 60% of maximum isometric pressure and progressed incrementally. The training protocol consisted of 30 repetitions three times daily.

Fourth, an individualized exercise protocol was developed for each patient based on identified biomechanical deficits. Exercises included Mendelsohn maneuver for patients with reduced laryngeal elevation, effortful swallow for those with decreased tongue base retraction, supraglottic swallow for patients with impaired airway protection, and Shaker exercise for those with reduced upper esophageal sphincter opening. Exercise selection and intensity were adjusted at monthly follow-up visits based on clinical progress.

#### 2.4.2 Standard care

Patients assigned to standard care received conventional swallowing rehabilitation as typically provided in clinical practice. This comprised general swallowing exercises including tongue range-of-motion exercises, lip strengthening exercises, and general effortful swallowing practice without instrumental biofeedback or electrical stimulation. Exercise instruction was provided by speech-language pathologists during the inpatient stay, with written instructions for home practice. Patients were seen at monthly intervals for progress monitoring and exercise reinforcement. The estimated daily training time in the standard care group was approximately 20–30 minutes.

Both groups received identical perioperative medical management including standardized drug therapy with betamethasone for edema reduction, ranitidine for stress ulcer prophylaxis, ambroxol for secretion management, cefazolin for infection prophylaxis, nadroparin for thromboprophylaxis, and nebulized salbutamol with trypsin for airway management.

### 2.5 Outcomes

The primary outcome was functional swallowing status measured by the Functional Oral Intake Scale (FOIS) at 6 months postoperatively. The FOIS is a validated 7-point ordinal scale ranging from 1 (nothing by mouth) to 7 (total oral diet with no restrictions), with higher scores indicating better function (24).

Secondary outcomes included FOIS scores at 1 and 3 months; MD Anderson Dysphagia Inventory (MDADI) composite scores at 1, 3, and 6 months (25); aspiration occurrence (defined as penetration-aspiration scale score ≥6 on videofluoroscopic evaluation) (26) at 1, 3, and 6 months; time to recovery of oral feeding (defined as time from surgery to achievement of FOIS ≥5); feeding tube dependency at 6 months; aspiration pneumonia incidence during the study period; and length of hospital stay. Protocol adherence in the intervention group was monitored through patient diaries and electronic device usage logs.

### 2.6 Statistical analysis

Sample size calculation was based on detecting a clinically meaningful difference of 0.5 points on the FOIS scale between groups at 6 months, assuming a standard deviation of 1.0, with 80% power at a two-sided significance level of 0.05. Accounting for an anticipated 15% dropout rate, a total sample size of 300 patients (150 per group) was required.

Analyses were conducted on an intention-to-treat basis. Given the ordinal nature of the FOIS, the primary outcome was analyzed using the Mann-Whitney U test, with results presented as median (interquartile range [IQR]). Effect size was quantified using r = Z/√N, where values of 0.1, 0.3, and 0.5 correspond to small, medium, and large effects, respectively. The Hodges-Lehmann estimator was used to calculate the median difference with 95% confidence interval. The MDADI composite score, although yielding a continuous summary measure (range 20–100), was also analyzed using the Mann-Whitney U test and reported as median (IQR) for consistency, given that it is derived from ordinal Likert-type items. Continuous variables were expressed as mean ± standard deviation or median with interquartile range as appropriate, and compared using independent samples t-tests or Mann-Whitney U tests. Categorical variables were expressed as frequencies with percentages and compared using chi-square tests or Fisher exact tests. The primary outcome was analyzed using an independent samples t-test, with effect size quantified using Cohen’s d. Relative risks with 95% confidence intervals were calculated for binary outcomes. Time-to-event analyses employed Kaplan-Meier methods with log-rank tests. Pre-planned subgroup analyses were conducted by tumor site, clinical stage (I–II vs III–IV), flap type, resection extent, and adjuvant therapy status. A sensitivity analysis excluding patients who received adjuvant (chemo)radiotherapy was performed to assess the robustness of the primary findings. All statistical analyses were performed using SPSS version 26.0 (IBM Corporation), with two-sided P values less than 0.05 considered statistically significant.

## 3 Results

### 3.1 Patient characteristics

Between January 2022 and June 2025, 358 patients were assessed for eligibility, of whom 300 met inclusion criteria and were randomly assigned to personalized rehabilitation (n=150) or standard care (n=150). Baseline characteristics were well balanced between groups (**Table 1**). The mean age was 57.66 ± 11.90 years in the intervention group and 58.23 ± 11.49 years in the control group (P = 0.673). Male patients predominated in both groups (68.7% vs 67.3%; P = 0.879). Tumor characteristics including site distribution, T stage, N stage, and clinical stage were similar between groups. The tongue was the most common primary site (40.0% vs 38.7%), followed by the floor of mouth (24.7% vs 26.0%). Anterolateral thigh flaps were the most frequently used reconstructive option (34.7% vs 35.3%), followed by radial forearm flaps (30.7% vs 29.3%). Baseline FOIS scores were comparable between groups (median, 4 [IQR, 3–4] vs 4 [IQR, 3–4]; P = 0.297), as were baseline MDADI composite scores (median, 50.90 [IQR, 42.59–59.86] vs 51.28 [IQR, 43.63–58.37]; P = 0.714).

**Table 1:** Baseline characteristics of patients.

Variable	Personalized rehabilitation (n = 150)	Standard care (n = 150)	P value
Age, years, mean +/- SD	58.90 ± 11.79	59.69 ± 12.34	0.569
BMI, kg/m2, mean +/- SD	23.79 ± 3.80	23.36 ± 3.41	0.295
KPS score, mean +/- SD	87.40 ± 8.47	87.80 ± 8.50	0.683
Baseline FOIS, median (IQR)	4.0 (3.0-4.0)	4.0 (3.0-4.0)	0.297
Baseline MDADI, median (IQR)	50.9 (42.6-59.9)	51.3 (43.6-58.4)	0.714
Sex, n (%)			0.439
Male	105 (70.0)	112 (74.7)	
Female	45 (30.0)	38 (25.3)	
Smoking status, n (%)			0.364
Never	33 (22.0)	43 (28.7)	
Former	76 (50.7)	66 (44.0)	
Current	41 (27.3)	41 (27.3)	
Diabetes, n (%)			0.765
Yes	26 (17.3)	29 (19.3)	
No	124 (82.7)	121 (80.7)	
Hypertension, n (%)			0.163
Yes	50 (33.3)	38 (25.3)	
No	100 (66.7)	112 (74.7)	
Tumor site, n (%)			0.091
Tongue	54 (36.0)	60 (40.0)	
Floor of mouth	33 (22.0)	40 (26.7)	
Buccal mucosa	34 (22.7)	16 (10.7)	
Palate	20 (13.3)	25 (16.7)	
Retromolar area	9 (6.0)	9 (6.0)	
T stage, n (%)			0.775
T1	24 (16.0)	26 (17.3)	
T2	55 (36.7)	47 (31.3)	
T3	42 (28.0)	48 (32.0)	
T4	29 (19.3)	29 (19.3)	
N stage, n (%)			0.729
N0	67 (44.7)	58 (38.7)	
N1	38 (25.3)	45 (30.0)	
N2	32 (21.3)	34 (22.7)	
N3	13 (8.7)	13 (8.7)	
Clinical stage, n (%)			0.804
I	8 (5.3)	11 (7.3)	
II	48 (32.0)	42 (28.0)	
III	57 (38.0)	57 (38.0)	
IV	37 (24.7)	40 (26.7)	
Flap type, n (%)			0.412
Anterolateral thigh	58 (38.7)	48 (32.0)	
Radial forearm	41 (27.3)	38 (25.3)	
Fibula	22 (14.7)	32 (21.3)	
Pectoralis major	18 (12.0)	16 (10.7)	
Local flap	11 (7.3)	16 (10.7)	
Tongue base involvement, n (%)			0.446
Yes	23 (15.3)	29 (19.3)	
No	127 (84.7)	121 (80.7)	
Oropharyngeal wall resection, n (%)			1.000
Yes	16 (10.7)	16 (10.7)	
No	134 (89.3)	134 (89.3)	
Soft palate resection, n (%)			0.610
Yes	22 (14.7)	18 (12.0)	
No	128 (85.3)	132 (88.0)	
Neck dissection, n (%)			0.129
Unilateral selective	59 (39.3)	71 (47.3)	
Unilateral modified radical	43 (28.7)	36 (24.0)	
Bilateral	24 (16.0)	30 (20.0)	
None	24 (16.0)	13 (8.7)	
Additional procedures, n (%)			0.944
Laryngeal suspension	5 (3.3)	5 (3.3)	
Cricopharyngeal myotomy	5 (3.3)	4 (2.7)	
None	140 (93.3)	141 (94.0)	
Adjuvant therapy, n (%)			0.270
None	50 (33.3)	62 (41.3)	
Radiotherapy	60 (40.0)	48 (32.0)	
Chemoradiotherapy	40 (26.7)	40 (26.7)	
Resection extent (tongue), n (%)			0.154
Partial glossectomy	12 (22.2)	10 (16.7)	
Hemiglossectomy	22 (40.7)	30 (50.0)	
Subtotal glossectomy	16 (29.6)	15 (25.0)	
Total glossectomy	4 (7.4)	5 (8.3)	

FOIS and MDADI baseline scores are reported as median (IQR). The following surgical and treatment variables have been added to the revised table and were balanced between groups (all P > 0.05): resection extent, tongue base involvement, oropharyngeal wall resection, soft palate resection, neck dissection type, additional procedures (laryngeal suspension, cricopharyngeal myotomy), and adjuvant therapy status.

BMI, body mass index; FOIS, Functional Oral Intake Scale; MDADI, MD Anderson Dysphagia Inventory; SD, standard deviation.

Among tongue cancer patients (n=114), resection extent included partial glossectomy (n=22, 19.3%), hemiglossectomy (n=52, 45.6%), subtotal glossectomy (n=31, 27.2%), and total glossectomy (n=9, 7.9%). Tongue base involvement was present in 52 patients (17.3%), oropharyngeal wall resection was performed in 32 patients (10.7%), and soft palate resection was required in 40 patients (13.3%). Neck dissection was performed in 263 patients (87.7%), including unilateral selective neck dissection (n=130, 43.3%), unilateral modified radical neck dissection (n=79, 26.3%), and bilateral neck dissection (n=54, 18.0%). Additional procedures included laryngeal suspension (n=10, 3.3%) and cricopharyngeal myotomy (n=9, 3.0%). Adjuvant radiotherapy was administered to 108 patients (36.0%) and adjuvant chemoradiotherapy to 80 patients (26.7%), while 112 patients (37.3%) received no adjuvant therapy. All surgical and treatment characteristics were well balanced between groups (all P > 0.05; **Table 1**).

### 3.2 Primary outcome

**Figure 2:**
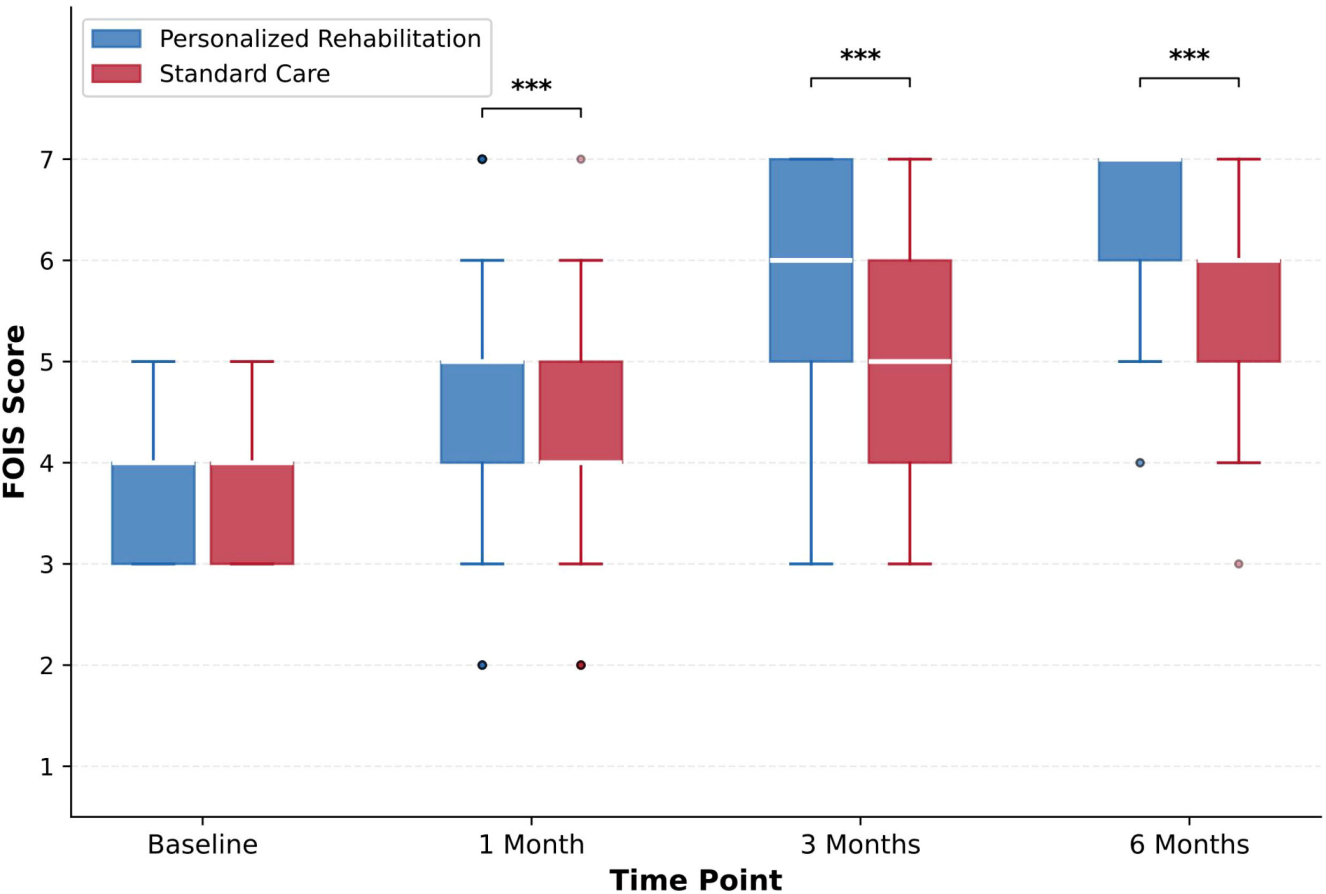
Functional Oral Intake Scale (FOIS) scores over time. Box plots show median (horizontal line), interquartile range (box), and range (whiskers). Asterisks denote statistical significance: ***P < 0.001.

At 6 months, patients in the personalized rehabilitation group achieved significantly higher FOIS scores compared with those receiving standard care (median, 7 [IQR, 6–7] vs 6 [IQR, 5–6]; Hodges-Lehmann median difference, 1.00; 95% CI, 1.00 to 1.00; P < 0.001; **Table 2**; **Figure 2**). The effect size was large (r = 0.366). In the intervention group, 82.0% of patients achieved FOIS scores of 6 or 7 (indicating oral diet with minimal or no restrictions), compared with 56.0% in the standard care group (P < 0.001).

**Table 2:** Primary and secondary outcomes.

Outcome	Personalized Rehabilitation (n = 150)	Standard Care (n = 150)	Hodges-Lehmann Difference (95% CI)	P value
Primary outcome				
FOIS at 6 months, median (IQR)	7.0 (6.0–7.0)	6.0 (5.0–6.0)	1.00 (1.00 to 1.00)	**<0.001**
Effect size r	0.366			
FOIS ≥6, n (%)	124 (82.7)	76 (50.7)		**<0.001**
Secondary outcomes				
FOIS at 1 month, median (IQR)	5.0 (4.0–5.0)	4.0 (4.0–5.0)	0.00 (0.00 to 1.00)	**<0.001**
Effect size r	0.184			
FOIS at 3 months, median (IQR)	6.0 (5.0–7.0)	5.0 (4.0–6.0)	1.00 (1.00 to 1.00)	**<0.001**
Effect size r	0.317			
MDADI at 1 month, median (IQR)	57.95 (48.71–67.60)	54.58 (45.35–62.70)		**0.045**
Effect size r	0.116			
MDADI at 3 months, median (IQR)	66.44 (58.83–76.52)	60.62 (53.70–69.20)		**<0.001**
Effect size r	0.217			
MDADI at 6 months, median (IQR)	75.57 (65.99–86.06)	65.80 (56.30–74.40)		**<0.001**
Effect size r	0.363			
Time to oral feeding, days, median (IQR)	15.84 (12.97–19.62)	19.80 (16.24–23.39)		**<0.001**
Hospital stay, days, median (IQR)	20.32 (16.69–25.13)	24.48 (19.84–29.03)		**<0.001**

All FOIS and MDADI outcomes are now reported as median (IQR) with Mann-Whitney U test P values and effect sizes (r = Z/√N). Hodges-Lehmann median differences with 95% CIs are provided for FOIS comparisons.

CI, confidence interval; FOIS, Functional Oral Intake Scale; IQR, interquartile range; MDADI, MD Anderson Dysphagia Inventory; SD, standard deviation.

Bold values indicate statistical significance.

### 3.3 Secondary outcomes

#### 3.3.1 Functional oral intake scale

Significant between-group differences in FOIS scores emerged as early as 1 month postoperatively (median, 5 [IQR, 4–5] vs 4 [IQR, 4–5]; P < 0.001; r = 0.184) and were maintained at 3 months (median, 6 [IQR, 5–7] vs 5 [IQR, 4–6]; P < 0.001; r = 0.317) and 6 months (**Figure 2**). The trajectory of recovery demonstrated progressively widening separation between groups across all time points, suggesting cumulative benefits of the personalized intervention.

#### 3.3.2 MD anderson dysphagia inventory

**Figure 3:**
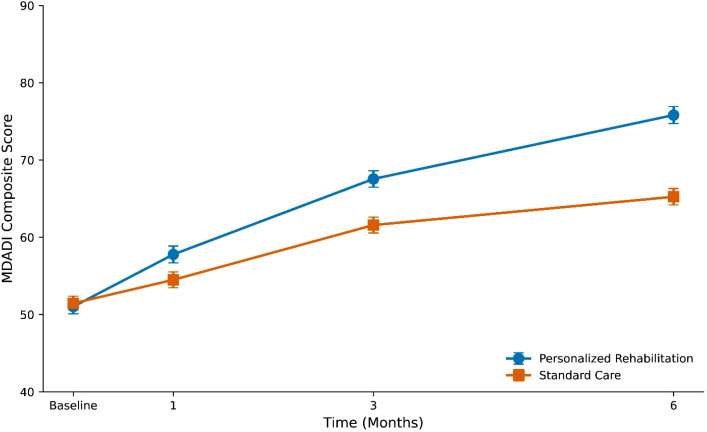
MD Anderson Dysphagia Inventory (MDADI) composite scores over time. Points represent mean scores; error bars indicate standard error of the mean. Significant between-group differences were observed at all follow-up time points (P < 0.05 at 1 month, P < 0.001 at 3 and 6 months).

Swallowing-related quality of life, measured by MDADI composite scores, was significantly higher in the intervention group at all follow-up time points (**Figure 3**). At 6 months, MDADI composite scores were median 75.57 (IQR, 65.99–86.06) in the personalized rehabilitation group versus median 65.80 (IQR, 56.30–74.40) in the standard care group (P < 0.001; r = 0.363). The observed difference exceeded the established minimal clinically important difference of 10 points for this instrument (27).

#### 3.3.3 Aspiration rates

**Figure 4:**
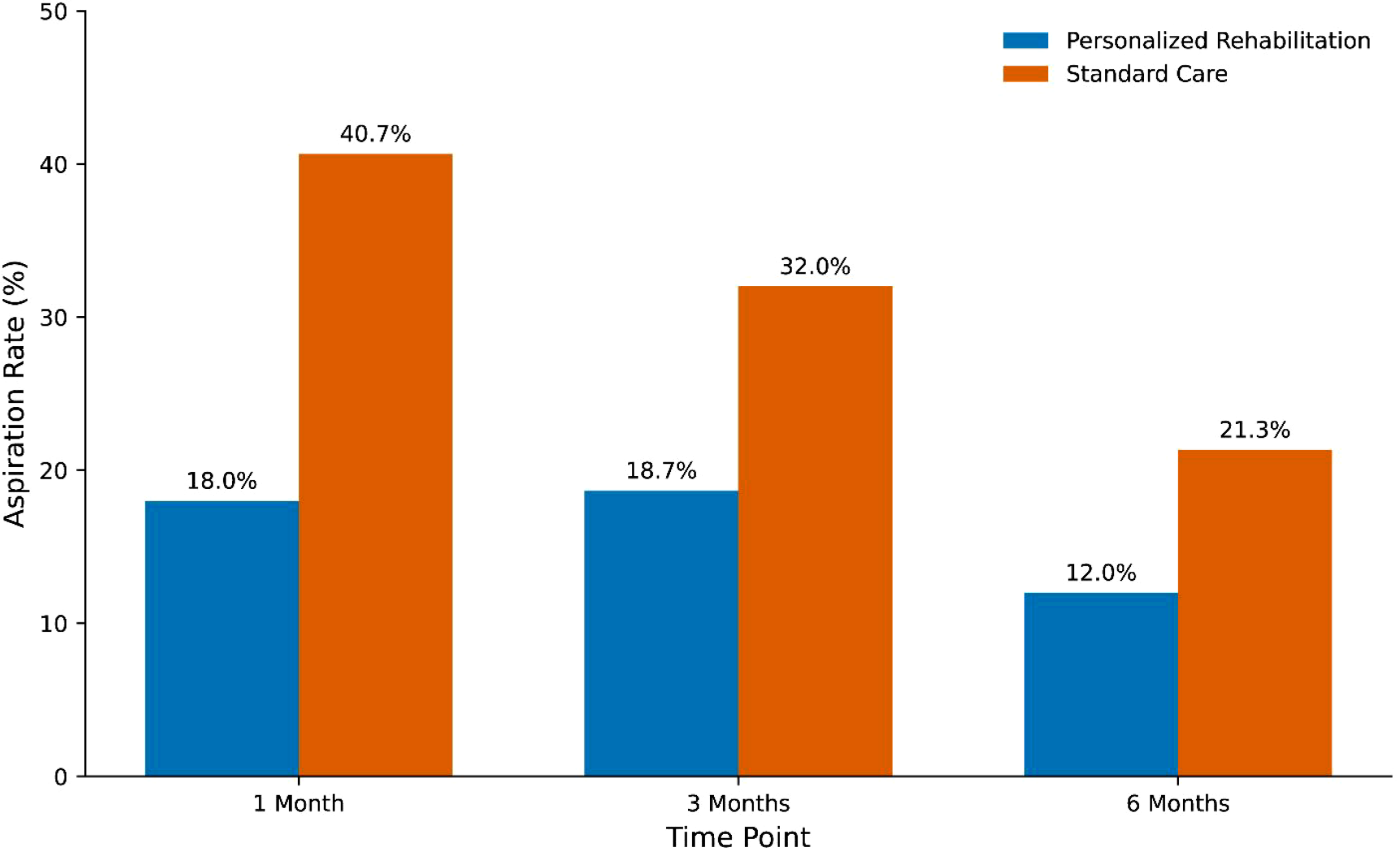
Aspiration rates at each follow-up time point. The personalized rehabilitation group demonstrated significantly lower aspiration rates compared with standard care at all time points.

Videofluoroscopic evidence of aspiration was significantly less frequent in the personalized rehabilitation group across all time points (**Figure 4**). At 1 month, aspiration was documented in 18.0% of intervention patients compared with 40.7% of controls (relative risk [RR], 0.44; 95% CI, 0.30 to 0.66; P < 0.001). At 3 months, rates were 18.7% versus 32.0% (RR, 0.58; 95% CI, 0.39 to 0.88; P = 0.012), and at 6 months, 12.0% versus 21.3% (RR, 0.56; 95% CI, 0.33 to 0.97; P = 0.044). The reduction in aspiration rates in the intervention group was most pronounced at 1 month, with sustained benefits throughout the follow-up period.

#### 3.3.4 Time to oral feeding recovery

**Figure 5:**
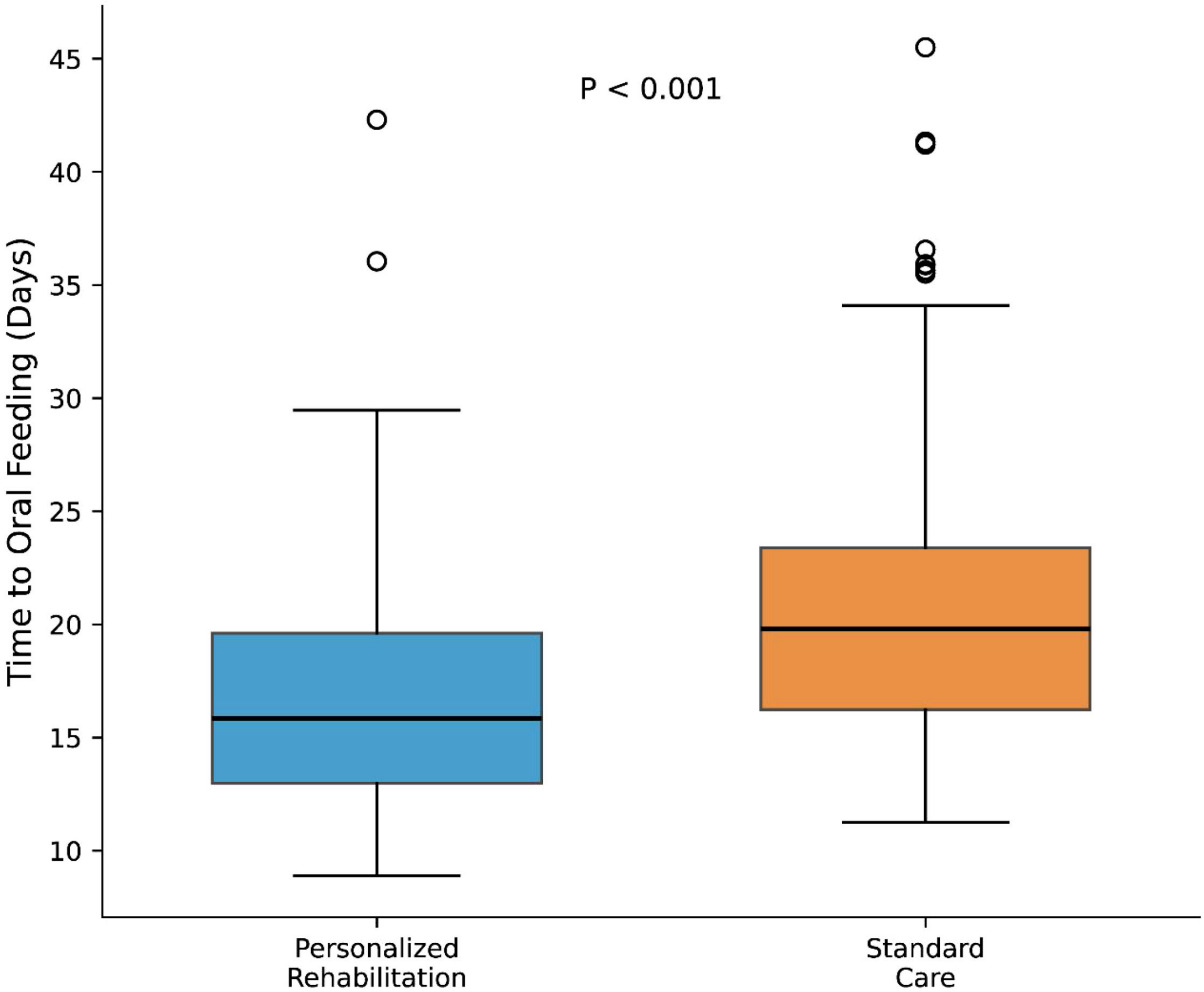
Time to recovery of oral feeding (days). Box plots show median (horizontal line), interquartile range (box), and range (whiskers). The personalized rehabilitation group achieved oral feeding significantly earlier than the standard care group (P < 0.001).

Patients in the personalized rehabilitation group achieved oral feeding (FOIS ≥5) significantly earlier than those in standard care. The median time to oral feeding was 15.84 days (interquartile range [IQR], 12.97-19.62) in the intervention group versus 19.80 days (IQR, 16.24-23.39) in the control group (P < 0.001; **Figure 5**). This represented a median reduction of approximately 4 days in the time required to resume meaningful oral intake.

#### 3.3.5 Feeding tube dependency and complications

At 6 months, feeding tube dependency was significantly lower in the personalized rehabilitation group (8.7% vs 28.7%; RR, 0.30; 95% CI, 0.17 to 0.55; P < 0.001; **Table 3**). Aspiration pneumonia occurred in 10.7% of intervention patients and 11.3% of controls, a difference that was not statistically significant (P = 1.000). Median length of hospital stay was 20.32 days (IQR, 16.69–25.13) in the intervention group and 24.48 days (IQR, 19.84–29.03) in the control group (P < 0.001).

**Table 3:** Aspiration rates and complications.

Outcome	Personalized (n=150)	Standard care (n=150)	RR (95% CI)	P
Aspiration at 1 mo, No. (%)	27 (18.0)	61 (40.7)	0.44 (0.30-0.66)	<0.001
Aspiration at 3 mo, No. (%)	28 (18.7)	48 (32.0)	0.58 (0.39-0.88)	0.012
Aspiration at 6 mo, No. (%)	18 (12.0)	32 (21.3)	0.56 (0.33-0.97)	0.044
Aspiration pneumonia, No. (%)	16 (10.7)	17 (11.3)	0.94 (0.50-1.78)	1.000
Tube dependency at 6 mo, No. (%)	13 (8.7)	43 (28.7)	0.30 (0.17-0.55)	<0.001

CI, confidence interval; RR, relative risk.

#### 3.3.6 Protocol adherence

Among patients in the personalized rehabilitation group, the median adherence rate was 80.01% (IQR, 72.29%–90.09%). Adherence was highest during the inpatient period (88.3%) and declined modestly during the outpatient phase (74.2%). No serious adverse events attributable to the rehabilitation interventions were observed.

#### 3.3.7 Subgroup and sensitivity analyses

**Figure 6:**
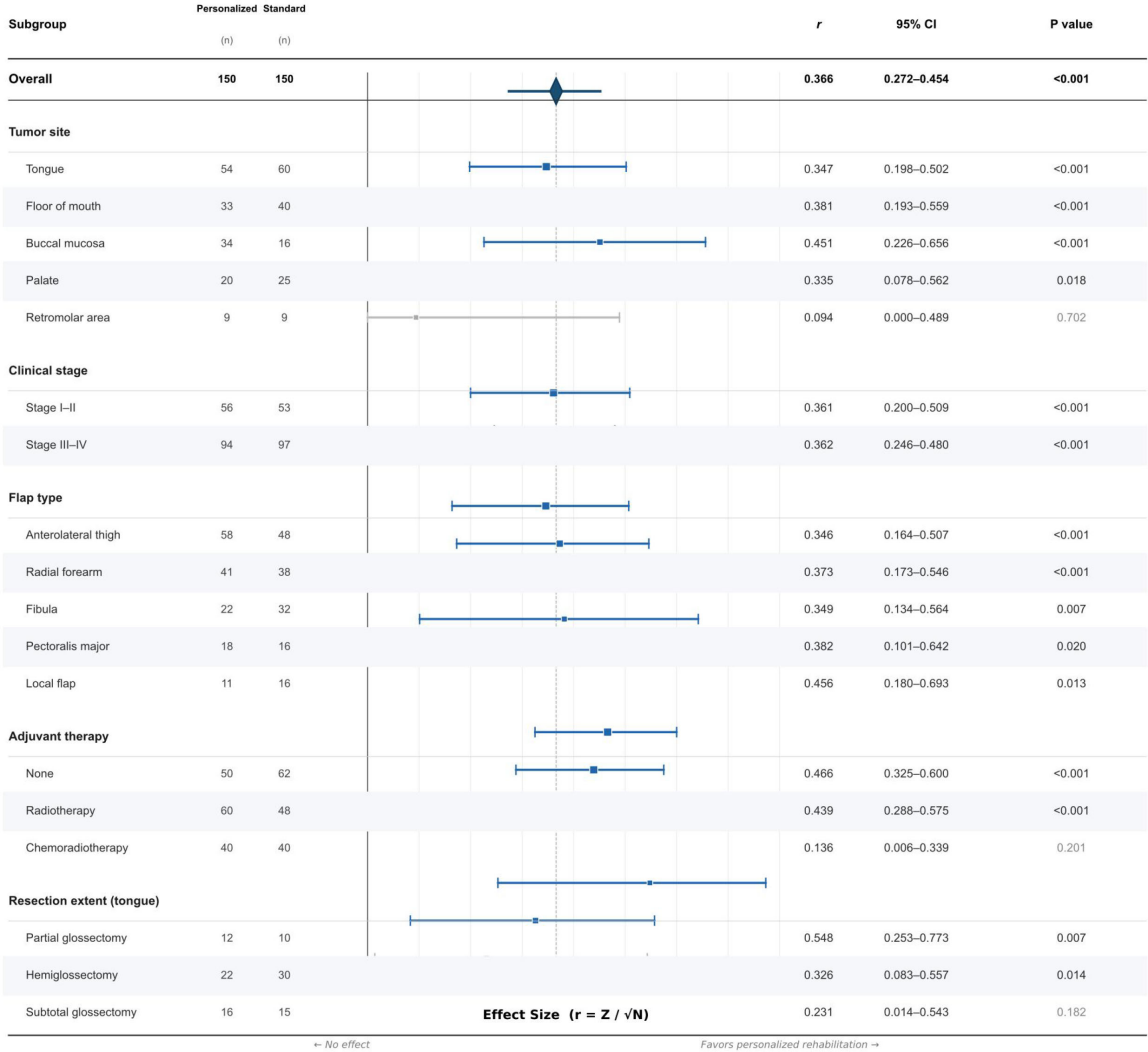
Forest plot of subgroup analyses for the primary outcome (FOIS at 6 months). Effect sizes (r) with 95% confidence intervals are shown for each subgroup. The dashed vertical line represents the overall effect size.

Subgroup analyses of the primary outcome (FOIS at 6 months) demonstrated that the benefits of personalized rehabilitation were generally consistent across tumor sites, clinical stages, and flap types (**Table 4**; **Figure 6**). By tumor site, significant between-group differences were observed for tongue (median, 6.5 vs 5.0; P < 0.001; r = 0.347), floor of mouth (median, 7.0 vs 6.0; P < 0.001; r = 0.381), buccal mucosa (median, 7.0 vs 5.0; P < 0.001; r = 0.451), and palate (median, 6.0 vs 6.0; P = 0.018; r = 0.335) subgroups. The retromolar area subgroup (n=18) did not reach statistical significance (P = 0.702), likely due to limited sample size.

**Table 4:** Subgroup analyses of the primary outcome (FOIS at 6 months).

Subgroup	Personalized Rehabilitation		Standard Care		Hodges-Lehmann		Effect	P
	*n*	Median (IQR)	*n*	Median (IQR)	Difference	95% CI	Size (r)	value
Tumor site								
Tongue	54	6.5 (6.0–7.0)	60	5.0 (5.0–6.0)	1.00	0.00 to 1.00	0.347	**<0.001**
Floor of mouth	33	7.0 (6.0–7.0)	40	6.0 (5.0–6.2)	1.00	0.00 to 1.00	0.381	**<0.001**
Buccal mucosa	34	7.0 (6.0–7.0)	16	5.0 (4.8–6.0)	1.00	0.00 to 2.00	0.451	**<0.001**
Palate	20	6.0 (6.0–7.0)	25	6.0 (5.0–6.0)	1.00	0.00 to 1.00	0.335	**0.018**
Retromolar area	9	6.0 (6.0–7.0)	9	6.0 (6.0–6.0)	0.00	−1.00 to 1.00	0.094	0.702
Clinical stage								
Stage I–II	56	7.0 (6.0–7.0)	53	6.0 (5.0–6.0)	1.00	0.00 to 1.00	0.361	**<0.001**
Stage III–IV	94	6.5 (6.0–7.0)	97	5.0 (5.0–6.0)	1.00	1.00 to 1.00	0.362	**<0.001**
Flap type								
Anterolateral thigh	58	6.5 (6.0–7.0)	48	6.0 (5.0–6.0)	1.00	0.00 to 1.00	0.346	**<0.001**
Radial forearm	41	6.0 (6.0–7.0)	38	5.0 (5.0–6.0)	1.00	0.00 to 1.00	0.373	**<0.001**
Fibula	22	7.0 (6.0–7.0)	32	5.0 (5.0–7.0)	1.00	0.00 to 2.00	0.349	**0.007**
Pectoralis major	18	6.5 (6.0–7.0)	16	5.5 (5.0–6.0)	1.00	0.00 to 1.00	0.382	**0.020**
Local flap	11	7.0 (6.0–7.0)	16	6.0 (5.0–6.2)	1.00	0.00 to 2.00	0.456	**0.013**
Adjuvant therapy								
None	50	7.0 (6.0–7.0)	62	5.5 (5.0–6.0)	1.00	1.00 to 1.00	0.466	**<0.001**
Radiotherapy	60	7.0 (6.0–7.0)	48	5.5 (5.0–6.0)	1.00	1.00 to 1.00	0.439	**<0.001**
Chemoradiotherapy	40	6.0 (5.0–7.0)	40	6.0 (5.0–7.0)	0.00	0.00 to 1.00	0.136	0.201
Resection extent (tongue)†								
Partial glossectomy	12	6.5 (6.0–7.0)	10	5.5 (5.0–6.0)	1.00	0.00 to 2.00	0.548	**0.007**
Hemiglossectomy	22	6.5 (5.2–7.0)	30	5.0 (5.0–6.0)	1.00	0.00 to 2.00	0.326	**0.014**
Subtotal glossectomy	16	6.5 (5.0–7.0)	15	6.0 (5.0–6.0)	0.00	0.00 to 1.00	0.231	0.182
Total glossectomy‡	4	—	5	—	—	—	—	—

CI, confidence interval; FOIS, Functional Oral Intake Scale; IQR, interquartile range.

Effect size r = Z/√N from Mann-Whitney U test. Values of 0.1, 0.3, and 0.5 correspond to small, medium, and large effects, respectively.

† Analysis restricted to patients with tongue tumors (n = 114).

‡ Total glossectomy subgroup (n = 9) too small for reliable estimation. — indicates insufficient sample size for analysis.

Bold values indicate statistical significance.

By clinical stage, the treatment effect was consistent for both early-stage (Stage I–II: median, 7.0 vs 6.0; P < 0.001; r = 0.361) and advanced-stage disease (Stage III–IV: median, 6.5 vs 5.0; P < 0.001; r = 0.362). By flap type, significant benefits were observed across all reconstruction methods, including anterolateral thigh flap (P < 0.001; r = 0.346), radial forearm flap (P < 0.001; r = 0.373), fibula flap (P = 0.007; r = 0.349), pectoralis major flap (P = 0.020; r = 0.382), and local flap (P = 0.013; r = 0.456).

Regarding adjuvant therapy, the personalized rehabilitation program showed significant benefits in patients who received no adjuvant therapy (median, 7.0 vs 5.5; P < 0.001; r = 0.466) and those who received adjuvant radiotherapy alone (median, 7.0 vs 5.5; P < 0.001; r = 0.439). However, in patients who received adjuvant chemoradiotherapy, the between-group difference did not reach statistical significance (median, 6.0 vs 6.0; P = 0.201; r = 0.136), suggesting that the concurrent toxic effects of chemoradiotherapy may attenuate the benefits of rehabilitation during the treatment period.

Among tongue cancer patients, subgroup analysis by resection extent demonstrated significant treatment effects for partial glossectomy (median, 6.5 vs 5.5; P = 0.007; r = 0.548) and hemiglossectomy (median, 6.5 vs 5.0; P = 0.014; r = 0.326). The subtotal glossectomy subgroup showed a trend toward improvement that did not reach statistical significance (median, 6.5 vs 6.0; P = 0.182; r = 0.231), and the total glossectomy subgroup (n=9) was too small for meaningful statistical comparison.

Sensitivity analysis excluding patients who received adjuvant (chemo)radiotherapy (n=112; intervention, n=50; control, n=62) confirmed the robustness of the primary findings. In this subgroup, the personalized rehabilitation group demonstrated significantly higher FOIS scores at 6 months (median, 7.0 [IQR, 6.0–7.0] vs 5.5 [IQR, 5.0–6.0]; P < 0.001; r = 0.466) and superior MDADI composite scores (median, 75.8 [IQR, 66.0–87.4] vs 66.1 [IQR, 56.9–74.6]; P < 0.001; r = 0.359).

## 4 Discussion

This randomized controlled trial demonstrates that a personalized swallowing rehabilitation program incorporating neuromuscular electrical stimulation, surface electromyography biofeedback, tongue pressure resistance training, and individualized exercise protocols significantly improves functional swallowing outcomes in oral cancer patients following surgical resection with free flap reconstruction. Compared with standard care, the intervention resulted in superior FOIS scores at 6 months, enhanced swallowing-related quality of life, reduced aspiration rates, accelerated recovery of oral feeding, and decreased feeding tube dependency. These findings provide robust evidence supporting the integration of multimodal, individualized rehabilitation approaches into routine postoperative care for this patient population.

The improvements observed across multiple outcome measures were clinically meaningful. The large effect size for the primary outcome (FOIS at 6 months) translated to a substantially higher proportion of patients achieving functional oral intake without significant restrictions, with direct implications for nutritional adequacy and quality of life. The improvement in swallowing-related quality of life, as measured by the MDADI composite score, exceeded the established minimal clinically important difference (27), indicating that patients in the intervention group experienced meaningfully better psychosocial outcomes encompassing social, emotional, and functional dimensions of well-being. The significant reduction in aspiration risk likely reflects the combined effects of strengthened pharyngeal musculature, improved coordination of the swallowing sequence, and enhanced sensory awareness achieved through the multimodal intervention approach. Additionally, the substantial reduction in feeding tube dependency at 6 months represents an important outcome from both patient and healthcare system perspectives, as prolonged tube feeding is associated with significant decrements in quality of life and increased healthcare costs (28).

Our findings extend the existing literature on swallowing rehabilitation in head and neck cancer populations, which has predominantly focused on patients receiving radiation-based treatment (13–15). The PRESTO trial and related studies have established the value of prophylactic swallowing exercises during radiotherapy, demonstrating that maintaining swallowing function during treatment prevents subsequent deterioration (14). However, surgical patients present fundamentally different rehabilitation challenges, requiring interventions that address immediate anatomical deficits rather than prevent progressive fibrosis. The present study provides evidence that multimodal, personalized rehabilitation is effective in this distinct population, filling an important gap in the evidence base.

The personalized nature of the intervention likely contributed to its efficacy. Rather than applying a uniform exercise protocol, the intervention tailored specific techniques to individual deficits identified through comprehensive baseline evaluation. For example, patients with reduced tongue base retraction received targeted effortful swallow training, while those with impaired laryngeal elevation focused on Mendelsohn maneuvers. A recent systematic review highlighted the need for such individualized approaches in dysphagia rehabilitation, noting that standardized protocols may be suboptimal for addressing the heterogeneous presentations observed in clinical practice (29). The integration of technology-assisted modalities further enhanced the program. Unlike previous studies applying NMES in isolation with mixed results (18–20), our approach combined electrical stimulation with active exercise within a comprehensive rehabilitation framework, with parameters individualized based on patient tolerance and response. The sEMG biofeedback component addressed known challenges with patient engagement by providing real-time performance feedback, potentially enhancing motor learning and exercise effectiveness (21).

Subgroup analyses demonstrated that the benefits of personalized rehabilitation were generally consistent across tumor sites, clinical stages, and reconstruction types, suggesting that the personalized approach is sufficiently flexible to address the specific functional consequences of different clinical scenarios. Patients with tongue tumors, who face particular challenges in bolus formation and propulsion, also derived significant benefit from the targeted tongue pressure resistance training using the IOPI device, consistent with findings by Barbera et al. (30) regarding variable functional outcomes depending on the reconstruction approach. Among tongue cancer patients, the treatment effect appeared larger in those with less extensive resections, while patients with subtotal glossectomy showed a non-significant trend, suggesting that more extensive resections may require more intensive rehabilitation. An important finding was the attenuated treatment effect in patients who received adjuvant chemoradiotherapy, likely attributable to the concurrent toxicities—including mucositis, xerostomia, and gastrointestinal symptoms—that may limit participation in rehabilitation exercises. The sensitivity analysis excluding patients who received adjuvant (chemo)radiotherapy confirmed that the primary findings remained robust.

Protocol adherence in the intervention group was notably higher than reported in many swallowing rehabilitation studies, where adherence rates of 13% to 64% are typical (31). This relatively high adherence likely reflects the incorporation of engaging technology-based components, regular follow-up with exercise protocol adjustments, and clear explanations of the rationale underlying each intervention component.

This study has several strengths, including its randomized controlled design, adequate sample size based on *a priori* power calculations, blinded outcome assessment, and comprehensive follow-up through 6 months. The use of validated outcome measures (FOIS, MDADI) enables comparison with other studies and facilitates translation to clinical practice. The detailed characterization of the intervention permits replication in other settings.

Several limitations should be acknowledged. First, the single-center design may limit generalizability to other healthcare settings. Second, the inability to blind patients and treating therapists to group allocation introduces potential performance bias, although this is inherent to rehabilitation research. Third, the 6-month follow-up period does not address longer-term outcomes or durability of treatment benefits. Fourth, the composite nature of the intervention, which included both different therapeutic modalities (NMES, sEMG biofeedback) and greater daily training time compared with standard care, precludes attribution of the observed benefits to any single component or definitive distinction between the effects of personalized content and training dose. Prior studies suggest that the effectiveness of NMES may depend on integration within a comprehensive framework rather than standalone application (18, 19), but dismantling studies and dose-matched designs are needed to clarify these contributions. Fifth, although subgroup analyses by adjuvant therapy status were informative, the study was not specifically powered to detect subgroup interactions. Future research should investigate the optimal intensity and duration of personalized programs, evaluate longer-term outcomes, assess cost-effectiveness, and explore emerging technologies such as mobile health applications for remote monitoring and exercise guidance (32).

## 5 Conclusions

A personalized swallowing rehabilitation program incorporating neuromuscular electrical stimulation, surface electromyography biofeedback, tongue pressure resistance training, and individualized exercise protocols significantly improves functional swallowing outcomes, reduces aspiration risk, and enhances swallowing-related quality of life compared with standard care in oral cancer patients following surgical resection with free flap reconstruction. These findings support the integration of multimodal, individualized rehabilitation strategies into routine postoperative management of this population. Implementation of personalized rehabilitation programs has the potential to reduce dysphagia-related morbidity, accelerate functional recovery, and improve patient quality of life following oral cancer surgery.

## Data Availability

The original contributions presented in the study are included in the article/supplementary material. Further inquiries can be directed to the corresponding authors.
